# A retrospective observational study – exploring food pantry referral as a clinical proxy for residents’ ability to address unmet health-related social needs

**DOI:** 10.1080/10872981.2024.2404295

**Published:** 2024-09-20

**Authors:** Michelle March, Daniel Schumacher, Andrew F. Beck, Mary Carol Burkhardt, Allison Reyner, Melissa Klein

**Affiliations:** a Division of General and Community Pediatrics, Cincinnati Children’s Hospital Medical Center, Cincinnati, OH, USA; b Division of Emergency Medicine, Cincinnati Children’s Hospital Medical Center, Cincinnati, OH, USA; c Department of Pediatrics, University of Cincinnati College of Medicine, Cincinnati, OH, USA; d James M. Anderson Center for Health Systems Excellence, Cincinnati Children’s Hospital Medical Center, Cincinnati, OH, USA

**Keywords:** Social determinants of health, food insecurity, medical education, Pediatrics, social accountability

## Abstract

**Background:**

Assessment of residents’ ability to address unmet health-related social needs to promote social accountability remains subjective and difficult. Existing approaches rely on self-assessment surveys of residents’ knowledge, skills, and attitudes following social determinants of health training, with few studies explicitly measuring clinical practice. We aimed to characterize social accountability using resident referrals to a food pantry embedded in a pediatric primary care center as an objective measure of resident ability to address unmet health-related social needs in clinical practice.

**Methods:**

This retrospective observational study occurred from 1 January 2019, to 30 June 2020, at an urban, pediatric primary care center with an embedded food pantry. All pediatric residents received social accountability education during a 2-week Advocacy rotation intern year. During clinic visits, pediatric residents were expected to act on results of a standardized social screen that included two food insecurity questions. Food pantry referral was the primary outcome. Food pantry referral data were extracted from food pantry logs.

**Results:**

During the 18-month study period, the pediatric primary care center food pantry was accessed at 1,031 visits. Of the 860 physician-based visits that resulted in pantry referral, 63% (*n* = 545) were initiated by residents. Eighty-six percent of residents (134/156) made ≥ 1 referral. Across all years, residents placed a mean of 3 (range 1–16) food pantry referrals.

**Conclusions:**

During our study, most residents placed at least one pantry referral in response to identifying food insecurity either via the screen or during conversation with the family. Referral to a primary care embedded food pantry, one way to address acute food insecurity may serve as a measurable proxy to assess residents’ ability to address unmet health-related social needs and promote social accountability in healthcare delivery.

## Background

Food insecurity (FI) affects one in seven children living in U.S. households and is associated with poor health and educational outcomes [1,2]. Pediatric institutions with resident continuity clinics are uniquely situated to screen for and mitigate health-related social needs, like FI. Children are seen frequently for well-child care and sick or follow-up visits. These continuity clinics often care for children and families from underserved and minoritized communities, the same children and families who disproportionately experience FI and related social determinants of health (SDH) [3]. The American Association of Medical Colleges, Accreditation Council for Graduate Medical Education and American Academy of Pediatrics promote the importance of a physician’s knowledge of SDH and underscore the need for education and assessment of trainees in this area [3–7]. Identifying effective ways for residents to apply SDH and social accountability education, and then have these skills assessed, enhances future practice. However, such assessments remain largely unexplored due to subjectivity and measurement difficulties [5,8]. Existing approaches often rely on self-assessment surveys of residents’ knowledge, skills, and attitudes following education. Few studies explicitly measure clinical practice [9], including actions to address identified health-related social needs, leaving a gap as medical education seeks to advance health equity through social accountability.

## Purpose

To characterize social accountability using resident referrals to a food pantry embedded in a pediatric primary care center (PPCC) as an objective measure of resident ability to address health-related social needs in clinical practice.

## Methods

This retrospective observational study occurred 1 January 2019, to 30 June 2020, at an urban PPCC in an academic medical center. The PPCC serves as the continuity clinic for a large pediatric residency program and medical home for 17,000 children (>30,000 visits annually). Most patients identify as Black (75%) and are publicly insured (85%).

**Figure 1.**
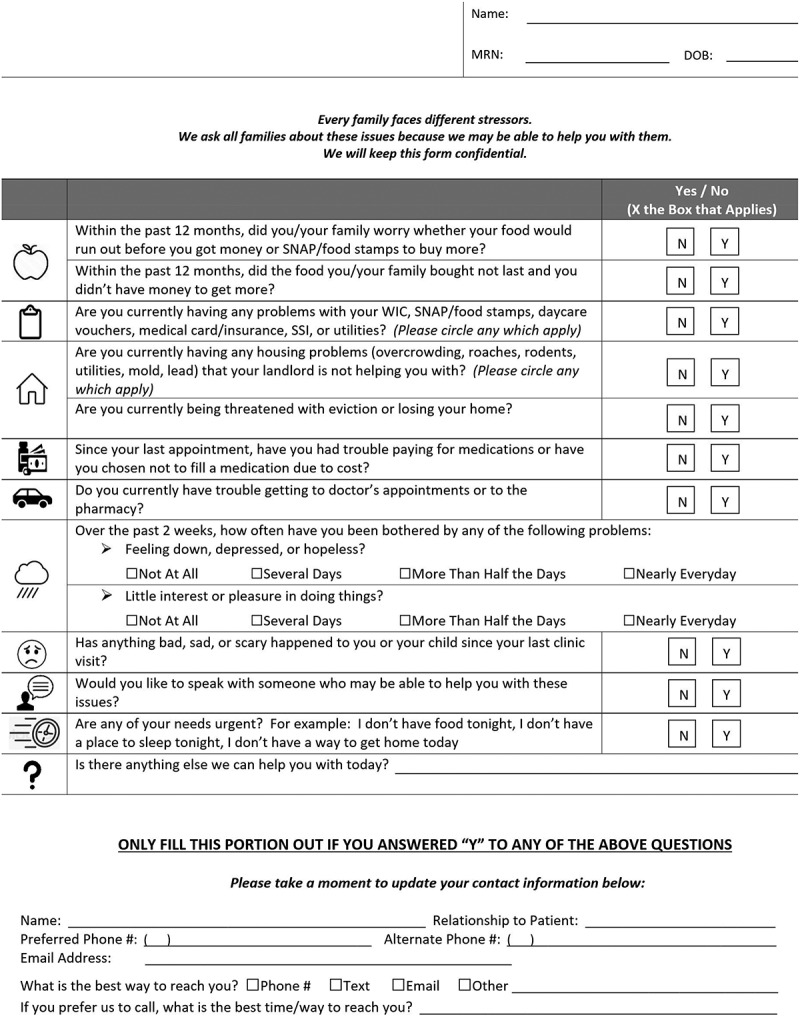
Standardized social screening questionnaire for patients and families.

All pediatric residents received social accountability education during a two-week Advocacy rotation intern year that includes detailed discussions of SDH, and commonly encountered health-related social needs, including FI. Pediatric residents were expected to act on results of a standardized social screen and discuss possible needs with families during clinical visits. The social screen included the 2-question Hunger Vital Sign [1,10], which identifies households at risk for FI (Figure 1). When FI was identified on the screen or during the visit, residents could complete a food pantry referral form, allowing real-time action to address acute FI by providing families with a 3-day supply of shelf-stable food.

All PPCC residents were included in this study and were sub-divided based on post-graduate year (PGY). Referral to the embedded food pantry was the primary outcome. Data from the referral form were entered into Research Electronic Data Capture (REDCap), a web-based, secure database, and then exported into R and Microsoft Excel to calculate descriptive statistics. This study was deemed exempt by the Institutional Review Board.

## Results

During the study, the PPCC food pantry was accessed at 1,031 visits. Data were excluded for seven visits with missing pantry logs and 156 pantry-only visits (food pantry used without clinical visit). Of the remaining 868 visits, 860 referrals to the food pantry were made by physicians and 8 by others (e.g., nurse practitioner, dietician). Of the 860 physician-directed referrals, 63% (*n* = 545) were initiated by residents despite residents seeing just 45% of total visits. Eighty-six percent of residents (135/156) referred to the food pantry at least once. Across all PGY years, residents placed a mean of 3 (range 1–16) food pantry referrals (Table 1).

**Table 1.** Resident referrals to the pediatric primary care center (PPCC) food pantry.

	Number of Pantry Referrals	Percentage of Total Physician Pantry Referrals	Mean and Range	Median and Interquartile Range
All Residents (*N* = 156)	545	63%	3 (1-16)	3
Post-Graduate Year 1 (*N* = 55)	198	23%	3 (1-16)	3 (4)
Post-Graduate Year 2 (*N* = 53)	147	17%	2 (1-9)	2 (3)
Post-Graduate Year 3 (*N* = 48)	200	23%	4 (1-12)	3.5 (5.5)

## Conclusions

During the study, most residents placed at least one pantry referral in response to identifying FI. A food pantry referral may allow residents to demonstrate their ability to apply social accountability education and respond to FI during clinical care. Use of resources like food pantries may serve as measurable clinical proxies to objectively assess resident ability to intervene and promote health equity. To our knowledge, this is the first study to explore using a clinically available, objective assessment for SDH and social accountability among residents.

Residency programs may consider exploring measurable outcomes of resident behaviors, such as referrals to embedded or co-located resources, as objective measures of residents’ skills addressing health-related social needs. Evaluating clinical practice outcomes, like pantry referrals, may serve as better indicators of skill development related to promotion of social accountability in healthcare delivery than previously reported metrics related to self-assessed knowledge, skills, and attitudes.

This study generated several learnings. First, we discovered the importance of having a robust process to track pantry referrals. We used paper referrals, which required manual data entry and subsequent pairing of pantry referrals with patient electronic health records (EHR). This was inefficient and susceptible to data errors. Following the study period, we upgraded to an EHR-based pantry referral which allowed for more efficient referral tracking. Second, we learned the importance of standardizing documentation within the EHR for resource provision when health-related social needs were identified. Third, it takes time for new resources to be utilized by providers during clinical practice and referral rates may increase in the future.

This study has limitations. First, it occurred at a single outpatient PPCC with many on-site resources. This may have impacted residents’ awareness of, and tendency to intervene on unmet health-related social needs. Second, an embedded food pantry may not be available elsewhere, limiting generalizability. Third, the study overlapped with the COVID-19 pandemic by four months, which could have affected referral patterns due to pandemic-related shifts in resource and financial constraints. Fourth, we could not validate FI presence and referral placement.

Next steps include understanding barriers faced by the residents who never referred to the food pantry and exploring ways to track resident performance in real-time and intervene when appropriate. While FI was our focus, future work could include establishing additional objective metrics for addressing other health-related social needs (e.g., referral to medical-legal partnership, psychology, social work, community health worker). Such interventions could serve as other ways of measuring effectiveness of social accountability education. Moreover, future studies could explore the utility of generating a social accountability score, summing residents’ referrals to pertinent resources. Such a score could help characterize referral patterns and serve as an entrustable professional activity focused on addressing unmet health-related social needs.

## Terms of consideration

This manuscript is our original work and has not been previously published in the same or substantially similar form previously, nor is it under consideration by any other print or electronic journal.
